# Transcranial magnetic stimulation over supramarginal gyrus stimulates primary motor cortex directly and impairs manual dexterity: implications for TMS focality

**DOI:** 10.1152/jn.00369.2023

**Published:** 2024-01-10

**Authors:** Nicholas P. Holmes, Nunzia Valentina Di Chiaro, Emily M. Crowe, Ben Marson, Karen Göbel, Dominykas Gaigalas, Talia Jay, Abigail V. Lockett, Eleanor S. Powell, Silvia Zeni, Arran T. Reader

**Affiliations:** ^1^School of Sport, Exercise and Rehabilitation Sciences, University of Birmingham, Birmingham, United Kingdom; ^2^School of Psychology, University of Nottingham, Nottingham, United Kingdom; ^3^Department of Psychology, University of Stirling, Stirling, United Kingdom

**Keywords:** corticospinal excitability, manual dexterity, mapping, movement, pegboard

## Abstract

Based on human motor cortex, the effective spatial resolution of transcranial magnetic stimulation (TMS) is often described as 5–20 mm, because small changes in TMS coil position can have large effects on motor-evoked potentials (MEPs). MEPs are often studied at rest, with muscles relaxed. During muscle contraction and movement, corticospinal excitability is higher, thresholds for effective stimulation are lower, and MEPs can be evoked from larger regions of scalp, so the effective spatial resolution of TMS is larger. We found that TMS over the supramarginal gyrus (SMG) impaired manual dexterity in the grooved pegboard task. It also resulted in short-latency MEPs in hand muscles, despite the coil being 55 mm away from the motor cortex hand area (M1). MEPs might be evoked by either a specific corticospinal connection from SMG or a remote but direct electromagnetic stimulation of M1. To distinguish these alternatives, we mapped MEPs across the scalp during rest, isotonic contraction, and manual dexterity tasks and ran electric field simulations to model the expected M1 activation from 27 scalp locations and four coil orientations. We also systematically reviewed studies using TMS during movement. Across five experiments, TMS over SMG reliably evoked MEPs during hand movement. These MEPs were consistent with direct M1 stimulation and substantially decreased corticospinal thresholds during natural movement. Systematic review suggested that 54 published experiments may have suffered from similar motor activation confounds. Our results have implications for the assumed spatial resolution of TMS, and especially when TMS is presented within 55 mm of the motor cortex.

**NEW & NOTEWORTHY** Transcranial magnetic stimulation (TMS) is often described as having an effective spatial resolution of ∼10 mm, because of the limited area of the scalp on which TMS produces motor-evoked potentials (MEPs) in resting muscles. We find that during natural hand movement TMS evokes MEPs from a much larger scalp area, in particular when stimulating over the supramarginal gyrus 55 mm away. Our results show that TMS can be effective at much larger distances than generally assumed.

## Graphical abstract

**Figure:**
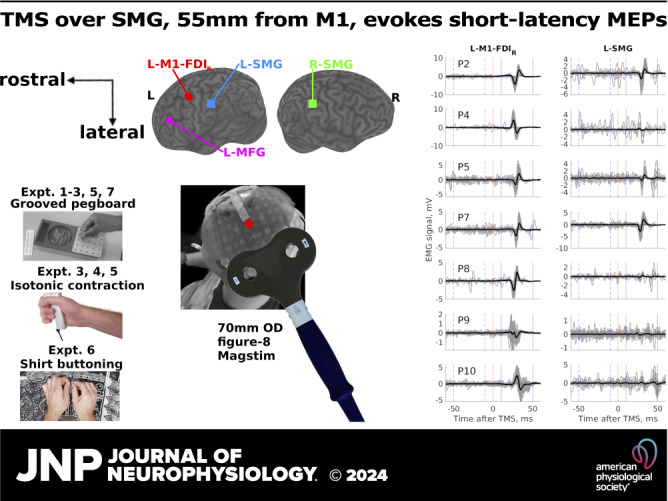

## INTRODUCTION

The human cortical motor system comprises primary (M1) and nonprimary (pre- and supplementary motor) areas in the frontal lobe, along with interconnected regions in the inferior (IPL) and superior (SPL) parietal lobules. M1 contains a topographical map of the body’s muscles (1). In general, the legs are represented superiorly and medially, the hands intermediately, and the face ventrally and laterally. The organization of the nonprimary motor areas is less topographic, but decades of studies in nonhuman and human primates have revealed effector-specific regions of parietal and premotor cortex (2), in particular for hand [e.g., anterior intraparietal area (AIP), Ref. 3] and eye [lateral intraparietal area (LIP), Ref. 4; frontal eye fields (FEF), Ref. 5] movements.

Area AIP in monkeys is thought to be homologous to a similar area in humans, the anterior intraparietal sulcus (aIPS; Ref. 6). Single neurons in AIP, and whole populations of neurons in aIPS, are implicated in the planning of hand prehension movements, particularly in object perception and prehension (7). Inactivation of AIP in monkeys causes deficits in shaping the fingers to grasp objects (8). In parallel, stimulating human aIPS with transcranial magnetic stimulation (TMS) has been used to study this area’s involvement in motor control (9). TMS provides a potentially powerful method to interfere with the planning and control of movement noninvasively, relatively safely, and with relatively low discomfort, depending on scalp location (10).

When planning the study presented here, and based on previous work in our laboratory (11, 12), we aimed to use TMS to examine the role of human supramarginal gyrus (SMG, the anterior portion of the inferior parietal lobule, lateral to area aIPS) in the control of manual dexterity. We reasoned that if the SMG is involved in finger shaping, object manipulation, and the planning and control of grasping movements (11, 13–15) then manual dexterity will be impaired when TMS is delivered. We used the grooved pegboard task as a difficult, well-studied manual dexterity task requiring accurate finger-and-thumb grasping and manipulation (e.g., Ref. 16).

As hypothesized, we successfully interfered with pegboard performance in our first two experiments, suggesting a role of the SMG in the control of manual dexterity. However, several participants reported the feeling that TMS directly evoked movements or “blocked” their hand and finger muscles during stimulation (17). We reasoned that these phenomena could be explained by a specific role of SMG in controlling, and perhaps commanding, movements either via direct connections between SMG and the corticospinal motor system or by neural connections to M1. Alternatively, interference with movement could occur because of an artifactual, remote, but direct stimulation of the hand area of primary motor cortex (M1-hand), without any intermediate neural connections from SMG. This latter hypothesis seemed very unlikely, given the broad agreement among TMS researchers that the spatial precision of TMS is ∼5–20 mm, at least for the motor cortex (see Ref. 18).

In eight experiments, a simulation of TMS-evoked electrical currents, and a systematic review, we characterized the effects of TMS, presented both near and far from the motor cortex, on corticospinal excitability during movement. We assessed a range of experimental contexts, from complete rest to complex bimanual coordination, with single or repetitive pulses of TMS presented over a wide area of the scalp and at one, four, or eight coil orientations. We recorded motor performance and motor-evoked potentials (MEPs). Our initial aim was to study the role of SMG in manual dexterity. In the end, our aim became to develop methods to distinguish between the real effects of TMS over SMG versus the artifactual effects of remote stimulation of M1-hand.

## METHODS

### Participants

A total of 84 healthy adults with no contraindications to TMS (19) participated in between one and six (mean = 1.38, SD = 0.83) experiments between February 2018 and August 2023 (Table 1). The experiments were approved by the University of Nottingham School of Psychology Ethics Committee (SoPEC; references: 708, 1040 R, F1018, F1053) and the University of Birmingham’s Science, Technology, Engineering and Mathematics Ethical Review Committee (ERN_18-2077AP13). Participants were recruited from and into the HandLab database, which in general comprises healthy adults from the local population of students and staff at the university. All participants gave written informed consent. No a priori power analyses were done. Rather, we work under the assumption that the TMS experiments were only worth doing, and the benefit/cost ratio only sufficiently high, when or if the effect sizes are large. In practice, the laboratory generally aims to recruit 12 participants per experiment and to replicate effects of interest across independent experiments and with converging sources of evidence. This sample size gives us ∼80% power to detect within-participant Cohen’s *d_z_* effect sizes of 0.8 or larger. Twelve participants per experiment is greater than the mean and median sample sizes (*N* ∼ 10) in the relevant experimental literature (see *Systematic Review: Studies with TMS near Motor Cortex during Hand Movement*). *Experiments 3* and *5* were interrupted for 2 years by the 2020–2022 COVID-19 pandemic, resulting in several small differences in recruitment and methods. Three participants began but did not complete an experiment because of feeling faint (*N* = 1, *experiment 4*), the pandemic (*N* = 1, *experiment 5*), or having a very high resting motor threshold (*N* = 1, *experiment 7*). We do not record participant ethnicity.

**Table 1.** Participants, experimental design, and TMS parameters

Exp	Participants					Head Sizes		Tasks	Reps	Distance from Cz, cm			TMS								
	N	Female/Male^†^	Age, years	**Handedness^††^ L/A/R**	MRI	N-I	E-E			Area	Lateral	Anterior	**RMT MSO**	Intensity		Sites	Machine	Orient-ations	Hz	Pulses	Muscles
														%MSO	%RMT						
1	12	6/6	24.3 (5.8)	0/0/12	5	36.4 (1.8)	35.5 (1.7)	Pegboard	3	L-FDI	∼	∼	64.0 (6.9)	69.5 (6.6)	109 (2.0)	2	Rapid^2^, biphasic	45º	1	30	∼
										L-SMG	−9.2 (1.3)	−2.9 (1.0)									
										L-MFG	−7.6 (1.1)	6.6 (1.4)									
										None	∼	∼									
2a	12	4/8	28.5 (4.3)	0/0/12	11	35.8 (1.3)	35.5 (1.7)	Pegboard	8	L-FDI	∼	∼	59.8 (8.2)	64.8 (9.8)	108 (4.1)	2	Rapid^2^, biphasic	45º	2	60	∼
										L-SMG	−9.2 (1.0)	−3.5 (1.4)									
										R-SMG	9.2 (1.6)	−2.5 (0.9)									
										None	∼	∼									
2b	7	1/6	29.0 (5.3)	0/0/7	6	36.4 (1.8)	36.1 (1.5)	Pegboard	1	L-FDI	−4.6 (0.4)	0.2 (0.8)	60.1 (9.8)	64.1 (10.6)	107 (5.3)	2	Rapid^2^, biphasic	45º	2	60	Thenar_R_
										L-SMG	-9.0 (1.4)	−3.4 (1.4)									FDI_R_
										R-SMG	9.5 (1.3)	−2.7 (0.7)									FDS_R_
																					EDC_R_
3	12	7/5	28.4 (7.3)	0/1/11	12	36.7 (1.4)	36.8 (1.4)	Pegboard	1	L-FDI	−5.1 (0.7)	0.1 (0.7)	58.3 (9.4)	63.0 (7.8)	109 (5.9)	5	BiStim^2^,	0º	1–2	10	Thenar_R_
								Isotonic		pos 2	−6.1 (0.4)	−0.8 (0.8)					monophasic	45º			FDI_R_
								Rest		pos 3	−7.1 (0.4)	−1.6 (0.9)						90º			FDS_R_
										pos 4	−8.2 (0.7)	−2.5 (1.1)						135º			EDC_R_
										L-SMG	−9.2 (1.0)	−3.4 (1.3)						180º			
																		225º			
																		270º			
																		315º			
4	12*	9/3	22.4 (4.1)	1/2/9	1	36.6 (1.2)	36.4 (1.3)	Isotonic	1	L-FDI+	−5.4 (0.6)	0.1 (0.6)	53.5 (6.4)	61.6 (6.4)	115 (5.6)	16	BiStim^2^,	45º	0.2	10	FDI_R_
																	monophasic	135º			ADM_R_
																		225º			FDS_R_
																		315º			DM_R_
5	12**	3/9	24.4 (6.3)	0/0/12	5	37.0 (1.4)	37.4 (1.3)	Pegboard	1	L-FDI+	−5.2 (0.7)	0.5 (0.8)	60.6 (12.2)	67.8 (10.8)	113 (8.6)	27	BiStim^2^,	45º	0.2	10	FDI_R_
								Isotonic									monophasic	135º			ADM_R_
																		225º			FDS_R_
																		315º			DM_R_
6	12	7/5	26.1 (8.6)	1/0/11	0	37.5 (1.1)	37.0 (1.3)	Buttoning	1	L-FDI+	−5.3 (0.6)	-0.1 (0.7)	68.7 (7.5)	68.8 (7.4)	100 (0.0)	27	BiStim^2^,	45º	0.2	10	FDI_L_
								Flanker									monophasic				FDS_L_
																					FDI_R_
																					FDS_R_
7	11***	7/4	29.4 (3.21)	1/0/10	2	37.4 (2.2)	37.9 (1.8)	Pegboard	1	L-FDI	−5.2 (0.3)	0.8 (0.4)	51.6 (8.6)	16-90	∼50-160	2	BiStim^2^,	45º	0.2	20	FDI_R_
								Rest		L-SMG	−8.7 (0.3)	−2.7 (0.4)					monophasic				ADM_R_
																					FDS_R_
																					EDC_R_
8	22	10/12	28.1 (7.9)	1/0/21	22	36.8 (2.3)	36.8 (1.3)	Neuronavigation	1	L-FDI	−5.9 (0.5)	0.0 (0.8)	62.2 (7.8)	68.4 (8.6)	110 (0.0)	27	∼	∼	∼	∼	∼
										L-SMG	−9.3 (0.8)	−3.4 (0.9)									

Numbers show counts, mean (SD), or degrees. *N*, number. Handedness: A, ambidextrous; L, left; R, right. ADM, abductor digiti minimi; DM, deltoid middle; EDC, extensor digitorum communis; E-E, distance between the 2 preauricular points; Exp., experiment; FDI, first dorsal interosseus; FDS, flexor digitorum superficialis; L-, left hemisphere; -l, left hand; MRI, magnetic resonance imaging T1 scan available; MSO, maximum stimulator output; N-I, distance from nasion to inion; Pulses, TMS pulses per condition and location and orientation; R-, right hemisphere; -r, right hand; Reps, repetitions per task and transcranial magnetic stimulation (TMS) combination; RMT, resting motor threshold. ∼Not applicable, or no data are available. *One additional participant was excluded for feeling faint. **One additional participant did not complete the experiment because of a public health pandemic response. ***One additional participant did not complete the experiment because of a RMT > 70%MSO. †Participants were only provided 2 options to describe their sex (female/male). ††Handedness was self-reported.

### Apparatus

A 25-hole Grooved Pegboard (Lafayette Instruments, United States) and a patterned gray short-sleeved shirt (Primark, United Kingdom) were used for the manual dexterity tasks and two grip force transducers (AD Instruments, United Kingdom) for the isotonic contraction task. Experiments were run with MATLAB (MathWorks Inc., Natick, MA) and data collected with LabChart (AD Instruments) on PCs running Windows.

TMS was delivered with a Magstim Super-Rapid^2^ (*experiments 1* and *2*) or a BiStim^2^ (*experiments 3–7*) (Magstim, United Kingdom) and one of two 70-mm-outer diameter figure-of-eight polyurethane-coated coils. One coil was flat; the other was a “branding iron.” Neuronavigation (*experiments 1*, *2*, *3*, *5*, and *8*) used Brainsight (Rogue Research, Canada).

Electromyography (EMG) was acquired with a PowerLab 16/30 and two Dual BioAmps (AD Instruments) with disposable circular silver/silver chloride electrodes positioned on the lightly abraded, cleaned (and shaved where necessary) skin over the target muscles. EMG data were sampled at 2–10 kHz and band-pass filtered online at 10–500 Hz. Grip force data were monitored in *experiments 3* and *4*, to ensure task compliance only: data were not analyzed.

### Design

**Figure 1.**
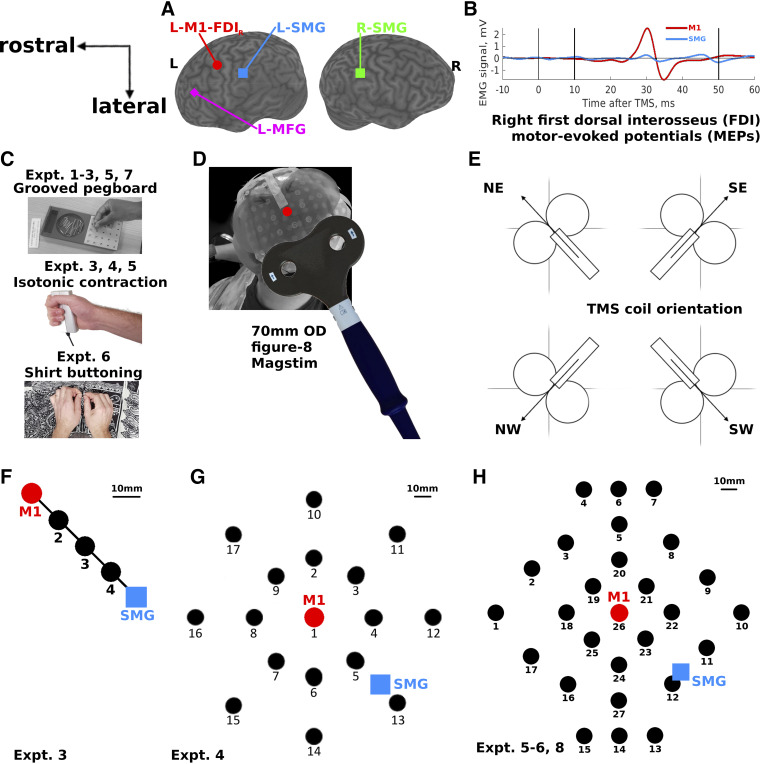
Transcranial magnetic stimulation (TMS) over the left motor cortex (L-M1-FDI_R_, red circles), left and right supramarginal gyrus (L-SMG, blue squares; R-SMG, green square), left middle frontal gyrus (L-MFG, magenta diamond), and 3, 16, and 26 other scalp locations (black circles). *A*: the cortical locations targeted in *experiments 1–3*. *B*: example averaged motor-evoked potentials (MEPs) recorded from 60 TMS pulses in a single participant’s surface electromyography (EMG) over the right hand first dorsal interosseus (FDI) muscle (*experiments 2b–7*). The vertical lines show the TMS onset (0 ms) and the MEP analysis window between 10 ms and 50 ms. *C*, *top*: the Grooved Pegboard task used in *experiments 1–3*, *5*, and *7*. *Middle*: the isometric contraction task used in *experiments 3*, *4*, and *5*. *Bottom*: the shirt-buttoning task used in *experiment 6*. *D*: the 27-location map (*H*), as seen from behind and to the left of 1 participant. The cloth map has 27 numbered targets. A transparent plastic TMS coil template is fixed over the central location (red dot, M1, *location 26*), and a Magstim 70-mm-outer diameter (OD) figure-8 coil is shown in the approximate “North-East” orientation, with handle 45° to the midline, pointing posterolaterally. Figures 4, 6, 7, and 9 show data from this perspective. *E*: 4 of the 8 coil orientations used in the study. Circles represent the coil wings; the rectangle represents the coil handle; arrows represent the direction of induced current flow in the brain. Coil orientations were labeled according to compass directions of the induced current flow in the brain, where North (N) is toward the nasion; East (E) is toward the right preauricular point; South (S) is toward the inion; and West (W) is toward the left preauricular point. *F*: the 5 locations stimulated in *experiment 3*, from L-M1-FDI_R_ to L-SMG in equidistant intervals; 10 mm scale bar reflects the average across participants. *G*: the 17 locations stimulated in *experiment 4*, with L-M1-FDI_R_ in the center (*1*) and L-SMG approximately midway between *locations 5* and *13*. *H*: the 27 locations stimulated in *experiments 5* and *6* and measured in *experiment 8*. The central location is L-M1-FDI_R_ (*26*), and L-SMG is partway between *locations 12* and *11*.

All experiments followed a within-participant repeated-measures design in which the main conditions and TMS locations were performed by all participants in a fully counterbalanced and/or pseudorandomized order (Table 1). The manipulated variables were task [pegboard, isotonic contraction, buttoning, reaction time (RT), rest], TMS location [left or right anterior SMG; left medial frontal gyrus (MFG); primary motor cortex (M1); and 3, 15, or 26 locations around M1], and TMS coil orientation (1, 4, or 8 orientations relative to the midsagittal sulcus, identified, following Meteyard and Holmes (10), with the compass directions North, North-East, East, South-East, South, South-West, West, North-West; Fig. 1*E*). The measured variables were behavioral (pegs placed, buttons buttoned, RTs) and electrophysiological (EMG activity in mV, mean MEP peak-to-peak amplitude in mV, mean MEP latency in ms).

### Procedure

#### General.

Participants were provided an information sheet, a TMS safety questionnaire, a consent form, and a TMS follow-up form. Background and instructions were provided verbally and on paper, questions were answered, and written informed consent was taken by an experimenter. Preparation for each experiment involved finding the scalp locations to stimulate with TMS, finding resting (and, for *experiment 7*, active) motor thresholds (RMT, AMT), and, for *experiments 2b–7*, attaching electrodes over the target muscles. Experiments involved repeating each task between one and eight times for each TMS location and coil orientation. Five tasks were used: *1*) Rest, *2*) Pegboard, *3*) Isotonic contraction, *4*) Shirt buttoning, and *5*) Flanker (details below). Experiments were designed so that each session lasted a maximum of 3 h. *Experiments 1–7* involved a total of between 120 and 2,160 TMS pulses per participant (mean = 795). *Experiments 2b–7* involved between 480 and 8,640 recorded EMG epochs per participant. Supplemental Methods S2 and Supplemental Table S1 provide the details. Two experimenters were present throughout, one holding the coil on the participant’s head while allowing the participant to perform the movement tasks and the other monitoring and recording the data. *Experiment 5* involved two sessions separated by a mean (SD) of 1.3 (0.5) days. Brief descriptions are provided in *Experiments and hypotheses*; details are in Table 1.

#### TMS: Motor cortex location, L-M1-FDI_R_.

Each participant’s head was measured from nasion to inion and between each ear’s preauricular point, intersecting at the vertex (Cz). We first used prior information about the likely location of the left M1-hand area (50 mm lateral and 10 mm anterior to the vertex; Ref. 20) to position the TMS coil. Several test pulses [e.g., at 50%, 60%, and 70% of the maximum stimulator output (MSO)] were used to elicit visible twitches in the right hand (*experiments 1* and *2*) or an MEP in the right first dorsal interosseus (FDI) muscle (*experiments 3–7*). For *experiments 1–5* and *7*, the coil was then moved around the likely optimal location until twitches or MEPs seemed to be maximal. For *experiments 5* and *6*, a grid of 20 or 25 locations with 10 × 10-mm spacing was oriented along the interaural axis, centered 50 mm left and 10 mm anterior to the vertex. Each point of the grid was stimulated five (*experiment 5*, *participants 1–6*, 20 locations) or two (the remaining participants, 25 locations) times, proceeding along the grid anteriorly to posteriorly, from the most medial row to the most lateral, while EMG was monitored from the right FDI. The location with the largest mean MEP was defined as the optimal location, L-M1-FDI_R_. Occasionally, two locations produced large mean MEPs. The chosen location was then either the approximate midpoint or the more central location (i.e., biased toward the prior population average). In line with much research, we used a standard ∼45° orientation for the coil handle when searching for the optimal L-M1-FDI_R_ location and did not also attempt to optimize the coil orientation (Supplemental Methods S5 and S7).

#### TMS: Motor thresholds and test intensity.

We used either an approximate (*experiments 1* and *2*; ∼5/10 visible twitches in 10 consecutive pulses, RMT_twitch_), the Rossini et al. (21) method (*experiments 3–5* and *7*; ∼5/10 MEPs > 50 μV peak to peak, RMT_Rossini94_), or a custom automated staircase method using QUEST (*experiment 6*; RMT_QUEST150_) to estimate RMT for each participant’s L-M1-FDI_R_. TMS intensity was then set at a mean of between 100% and 115%RMT across the six experiments, with most experiments setting the intensity close to 110%RMT (Table 1). Since the hardware required to run the QUEST method for *experiment 6* added some noise to the raw EMG data, RMT_QUEST150_ was estimated using a threshold of 150 μV. This resulted in significantly higher estimates of RMT, but as we stimulated at 100%RMT_QUEST150_ in *experiment 6*, the intensity of TMS measured with %MSO did not differ significantly between any two experiments (*experiments 1–6*, all *P* > 0.05, Bonferroni corrected for 21 comparisons). It is not possible to stimulate at exactly 110% RMT because the Magstim equipment does not produce fractional intensities. Furthermore, we occasionally have to lower the intensity because of participant discomfort and/or coil overheating. Further detail is provided in Supplemental Methods S4.

In *experiment 7*, AMT was determined during the pegboard task, with TMS delivered by the experimenter, approximately during grasping or placing of a peg. Ten trials’ EMG data were averaged, and AMT_pegboard_ was the lowest intensity that resulted in either ∼5/10 MEPs or the average across MEPs having a peak-to-peak amplitude of above 50 μV (typically, ∼100–200 μV). Perhaps because of improved signal to noise in the EMG system after moving to a new laboratory, or different TMS hardware and pulse waveforms, 110%RMT_Rossini94_ in *experiment 7* [mean (SD) = 56.8 (9.5)%MSO] was significantly lower than that used in *experiment 1* [69.5 (6.6)%MSO, *t*(21) = 3.74, *P* = 0.001]. RMT varies substantially across hardwares, coils, waveforms, and participants (e.g., see Ref. 22, particularly their Fig. 3D); we discuss this in Supplemental Methods S6.

#### TMS: Site selection.

*Experiments 1–4* targeted the anterior portion of the left SMG. When an individual structural MRI was available, this was done using the gross anatomy for anterior parietal cortex, guided by the coordinates MNI[−57, −44, 44] mm from a previous study (12). When no MRI scan was available, the mean scalp coordinates from previous participants as recorded in the HandLab participant database, LabMan, were used. These coordinates lateral and anterior to vertex have small standard deviations, across individual neuronavigated brain and scalp measurements, of 7–16 mm.

In *experiment 1*, the control location was the left MFG. This was selected with https://tms-smart.info as a strong control for TMS-related pain, twitches, and annoyance (10). Anecdotally, participants in *experiment 1* reported more pain and discomfort for the L-MFG than for the L-SMG site, so the control for *experiment 2a* was changed to R-SMG. *Experiment 3* used L-M1-FDI_R_, L-SMG, and three intermediate locations equally spaced along a straight line between L-M1-FDI_R_ and L-SMG. *Experiments 4–6* stimulated a set of 16 or 27 locations arranged in a grid, centered on each participant’s optimal location for L-M1-FDI_R_. *Experiment 7* stimulated L-M1-FDI_R_ and a location 55 mm away, over L-SMG, at a range of seven stimulation intensities.

#### TMS: Coil orientation.

For *experiments 1*, *2*, *6*, and *7*, the coil was held in a “standard” orientation for motor cortex, with the coil handle pointing ∼45° posteriorly and laterally away from the midsagittal plane so that the electrical current ran anteromedial to posterolateral and the induced current in the brain posterolateral to anteromedial. Initially, there was no specific reason for choosing this orientation, given that the optimal orientation for stimulating the target area of SMG is unknown, and in *experiments 1* and *2* the Magstim Rapid^2^ stimulator produced biphasic TMS pulses. *Experiments 3–7* used the BiStim’s monophasic stimulation, and *experiments 3–5* manipulated coil orientation as a method of testing the orientation specificity of MEP responses. Following Meteyard and Holmes (10), the standard orientation of ∼45° to the midsagittal plane was termed “North-East” (with the coil handle pointing posterolaterally, toward “South-West”). This orientation was used in all experiments. *Experiment 3* tested eight coil orientations at ∼45° intervals. *Experiments 4* and *5* tested four orientations at ∼90° intervals.

### Experiments and Hypotheses

The details of all participants and experiments are given in Table 1. The following provides a brief description of each experiment.

#### Experiments 1 and 2a: Does TMS over L-SMG impair right hand manual dexterity?

*Experiment 1* tested the hypothesis that L-SMG is involved in right hand dexterity. Participants performed the pegboard task while TMS was applied at 1 Hz over L-SMG and over L-MFG, a control location that was selected for its subjective annoyance and discomfort (Fig. 1, *A* and *C*), and when held away from the head. For all experiments, the movement tasks continued until all the TMS pulses had been presented. In *experiments 1* and *2a*, participants were asked to place as many pegs as they could within 30 s, starting with the first TMS pulse and stopping when the experimenter said “stop” or removed the TMS coil from the head. *Experiment 2a* was the same as *experiment 1*, except that TMS was applied at 2 Hz, the control location was R-SMG (Fig. 1, *A* and *C*), and more task repetitions were performed.

#### Experiments 2b and 3: Does TMS over L-SMG evoke MEPs?

After the behavioral results of *experiments 1* and *2*, additional EMG data were collected to test the hypothesis that TMS over nonmotor areas in the parietal lobe evokes MEPs. *Experiment 2b* was performed with a subgroup of participants who returned for this follow-up experiment in which 2-Hz TMS was applied over L-M1-FDI_R_ or L-SMG for 30 s each (i.e., 60 TMS pulses per location), during performance of the pegboard task and recording of EMG from four hand and arm muscles (Fig. 1*B*).

After we observed that TMS over SMG could induce MEPs, *experiment 3* tested the hypothesis that coil orientation, distance from L-M1-FDI_R_, and the movement task all significantly affect the mean MEP amplitude. Eight coil orientations were used, from 0° to 360° on polar axes at 45° intervals (Fig. 1*E*). Five locations were stimulated: L-M1-FDI_R_, L-SMG, and three equally spaced locations, calculated for each participant, between these two scalp areas (Fig. 1*F*). Three different motor tasks were used: pegboard, isotonic contraction in which participants continuously squeezed the dynamometer at ∼10% of maximum voluntary contraction, and rest. From previous work we had noted, anecdotally, that active motor thresholds seemed much lower during goal-directed movements like reaching and grasping (23). This motivated the comparisons between dynamic movement, isotonic contraction, and rest.

At each location, 10 pulses of TMS were presented at each of eight coil orientations. To reduce the overall duration of the experiment and to approximately match the frequencies of stimulation used in *experiments 1* and *2*, TMS pulses from the BiStim^2^ unit were given in pairs, separated by a pseudorandom interval of 500–999 ms (i.e., at 1–2 Hz), and pairs of pulses were given once every ∼5–10 s. Locations were tested in pseudorandomized order, and orientations were pseudorandomized within each location. Neuronavigation was used where a recent MRI scan was available. The predictions were that MEP amplitude will decrease monotonically with distance from L-M1-FDI_R_; MEP amplitude will show a significant coil orientation preference around the North-East direction whenever L-M1-FDI_R_ is being stimulated, and this orientation preference will decrease with distance from L-M1-FDI_R_ but remain significant for TMS over L-SMG; and MEP amplitude will be significantly larger, and MEPs will be evoked by TMS applied further away on the scalp, during active movement tasks (pegboard, isotonic contraction) compared to rest.

#### Experiments 4 and 5: Mapping MEPs across the scalp.

*Experiment 4* built upon the results of *experiment 3* by recording MEPs following TMS over L-M1-FDI_R_ and 16 surrounding locations arranged in two concentric circles of 35-mm and 70-mm radius, with the coil held at four orientations per location (Fig. 1*G*). The hypotheses were first, that MEPs would be evoked from the inner circle, 35 mm away, but perhaps not from the outer circle 70 mm away from L-M1-FDI_R_ and second, that locations where TMS produced MEPs would show the standard North-East coil orientation preference. Only a single motor task, isotonic contraction, was tested.

*Experiment 5* repeated *experiment 4* but increased the number of TMS locations to 27, allowing a more homogeneous coverage of the scalp. The two motor tasks were pegboard and rest (Fig. 1*H*). The hypotheses were that some scalp locations between 35 mm and 70 mm from L-M1-FDI_R_ would show significant MEP responses, there would be coil orientation preferences for North-East, and that the pegboard task would result in larger and more orientation-specific MEPs over a larger scalp area than the isotonic contraction task.

#### Experiment 6: Comparing a bimanual dexterity text with a bimanual reaction time task.

*Experiment 6* repeated *experiment 5*, but using a single coil orientation (North-East), without the rest condition and with two new bimanual tasks to test the generalizability of the effects from previous experiments: a bimanual dexterity task (repeatedly buttoning-up and unbuttoning a shirt) and a more “cognitive” bimanual RT task to report the color of a central target square while ignoring peripheral distractor squares (the colored version of the Flanker task; 46). Participants pressed a key with their left or right index finger as quickly and as correctly as possible after the appearance of the central target square, while trying to ignore the distractor squares presented 200 ms earlier.

#### Experiment 7: Can MEP latencies discriminate between cortical sources?

*Experiment 7* was conducted after discussion with an expert colleague to examine the latency of MEPs evoked from TMS over L-SMG. *Experiments 1–6* used the same TMS intensity for all stimulation sites per participant, regardless of distance from L-M1-FDI_R_. In *Experiment 7*, TMS intensity was varied separately for L-M1-FDI_R_ and L-SMG sites and for rest and pegboard tasks to acquire four MEP recruitment curves, each with seven intensity levels in steps of 5%MSO. The lowest intensity in the L-M1-FDI_R_ pegboard condition was 5%MSO below AMT_pegboard_ (e.g., 25, 30, 35, 40, 45, 50, 55%MSO); the L-M1-FDI_R_ rest condition minimum was 5%MSO below RMT_Rossini94_; the L-SMG condition minima were 5%MSO above RMT_Rossini94_. For participants with RMT_Rossini94_ ≥ 55%MSO, the ranges were shifted to keep the maximum intensity at 90%MSO. The experiment aimed to generate a wide range of MEP amplitudes and latencies for each condition and coil location. During this experiment we also returned to the data from *experiment 3*, to analyze the MEP latencies across all conditions, positions, and orientations.

#### Experiment 8: Neuronavigation and computer simulation.

We measured the scalp locations and MRI coordinates of 27 target sites on 22 participants’ heads. These sites covered the locations targeted in *experiments 3–7*. With the 22 MRI scans available from these participants, an electric field simulation was performed with SimNIBS 4.0.0 (24), modeling four coil orientations at each of 27 measured locations, with TMS at 110%RMT_Rossini94_ per participant, using the MagStim D70 coil model with maximum current change (d*I*/d*t*_max_) = 114.7 A/μs (model 13 in Ref. 22). 110% RMT_Rossini94_ was chosen based on the mean intensities used in our own work and on the results of the systematic review (see results). The simulation used the default values for connectivity and coil distance.

The mean simulated induced electric field (magn E) in the gray matter within a three-dimensional (3-D) voxel centered on L-M1-FDI_R_ (MNI[−38, −15, 58] mm, 11 × 11 × 11 mm; Ref. 25) was extracted and compared with the mean MEP amplitudes measured from the 27 locations and four orientations from *experiment 5* (i.e., a correlation between independent group means). The 27 locations per participant were transformed into MNI space, and the anatomical structures underlying each location were estimated by finding the nearest gray matter voxel to each location, projecting a vector from the TMS site through the nearest gray matter voxel and 6 mm into the brain. This brain voxel was used within FSL (26) to query the Harvard-Oxford and Juelich probabilistic atlases. For seven participants, we had both MEP and MRI data available. This allowed us to create a 3-D volume of the smoothed, interpolated mean MEP amplitude across the head and brain, by computing a weighted sum of MEPs, where the weight for each voxel in the image volume was proportional to the inverse of distance between the voxel and each of the 27 scalp locations. Using the open-source FieldTrip toolbox for MATLAB (27), we segmented each MRI scan and used the gray and white matter segments to generate a mask of the weighted MEPs. We thresholded these images at the mean weighted MEP, rescaled the remaining voxels to a range of 0 to 1, transformed each participant’s image to the MNI152 1 × 1 × 1-mm atlas, smoothed, and computed the mean weighted MEP image across participants. The purpose was to create an approximate visualization of which brain areas might result in hand motor activation confounds when targeted with TMS at 110%RMT_Rossini94_.

### Systematic Review of Closely Related Studies

A systematic review was conducted to find previous studies that might be affected by TMS proximity to M1-hand during hand or arm movement. The search string: “(TMS OR transcranial magnetic stimulation) AND (pariet* OR premotor) AND (reach* OR grasp* OR move*)” was entered into PubMed on 5th May 2023. Inclusion criteria were healthy adult participants, English language, TMS presented during active movement (excluding simple keyboard or mouse-button pressing movements), use of a single TMS coil (excluding twin- or triple-coil studies), TMS presented away from M1 but within ∼70 mm of M1, and hand or arm movements (excluding eye and foot movement studies).

We identified 660 results from a PubMed search and 190 papers retrieved from references and prior knowledge of potentially relevant articles. Sixty-eight duplicates were removed. Seven hundred eighty-two were screened on their title, resulting in 315 excluded. Four hundred sixty-seven were screened on the abstract, resulting in 159 excluded (for details, see the PRISMA flow chart, Supplemental Fig. S1). Three hundred eight full articles were screened in detail. The typical number of experiments per paper was a mean (SD) of 1.5 (1.2), median = 1. The 308 articles contained 418 separate experiments, of which 368 experiments were excluded based on two or more TMS coils (111), TMS before movement onset (97), M1 TMS or MEPs only (47), keyboard or mouse button presses (30), eye movements (22), repetitive (r)TMS or offline TMS (20), theta-burst TMS (17), TMS >70 mm from M1 (7), nonmotor task (7), resting (4), nonhand movement (4), testing only patients (1), or reporting only neuroimaging data (1). The 50 included experiments (from 22 articles) involved 25 grasping, 15 reaching, 5 finger tapping, and 5 more complex movements (drawing, writing, imitation). Details of excluded and included articles and experiments are in our repository, https://osf.io/2xytm.

The 50 reviewed experiments (total *N* = 518) presented single (13), double (12), triple (9), or more (16) pulses of TMS during hand and/or arm movements. Experimental groups included a mean (SD) of 9.8 (3.8) and a median of 10 participants, were mostly young [mean of mean (mean of SD) ages = 26.8 (4.0) yr], recruiting equally males (*N* = 224) and females (*N* = 223; *N* = 71 sex not reported). One study included a left-handed group (*N* = 8); all remaining studies involved only a single mixed-handed person and 384 right-handed people (*N* = 125 handedness not reported). Thirty-two experiments recorded EMG data from a mean (SD) of 1.3 (0.8) muscles (range 1 to 5). Thirty-two experiments included individual structural MRI data for participants. The mean (SD) TMS coil size was 71.5 (11.7) mm, and mean (SD) TMS intensity was 109 (14)%RMT. To assess the likely TMS locations in standard coordinates, if MRI coordinates were labeled “Talairach,” it was assumed they were using the MNI template, unless the authors reported using BrainVoyager or Softaxic, when the coordinates were converted to MNI space (https://bioimagesuiteweb.github.io/webapp/mni2tal.html).

### Analysis

#### Open data and analysis strategy.

All stages of data analysis, apart from measuring MEP latencies in *experiment 7*, were automated with custom MATLAB code. Data and code are freely available at our Open Science Framework page (https://osf.io/2xytm), and we will assist any researcher in reusing or reanalyzing our data. Over 5 yr, while developing analysis code and while generating and testing our hypotheses, many hundreds of graphical displays and statistical tests were looked at by the authors (primarily N.P.H.). These data previews have influenced the final choice of statistical analyses reported, some of which were selected over others. The same code was used for the final analyses and across-group hypothesis tests in all experiments. We are developing resources for sharing TMS methods and meta-analytic data (https://github.com/TMSMultiLab).

#### Behavior.

Behavioral data were analyzed with within-participant *t* tests and repeated-measures ANOVAs, with Greenhouse–Geisser corrections where needed, on the within-participant means per condition.

#### Electromyography.

When EMG was recorded, all four available channels (i.e., muscles) were used. For clarity and simplicity, only data from the right hand FDI muscle are reported in detail, since right FDI was used to calibrate TMS intensity and was recorded in all experiments (Table 1). All four muscles in each experiment were analyzed in the same way. Results for all muscles were similar and are available in our data repository. Offline, EMG data were filtered with a second-order dual Butterworth band-pass filter between 25 and 250 Hz and then segmented to 200 ms before and after the TMS pulse using 5-V TTL hardware triggers.

#### Motor-evoked potentials.

MEPs were measured as the peak-to-peak difference in EMG activity within a window of 10–50 ms after TMS. We did not attempt to measure MEP waveforms and relied instead on this very simple measure of EMG activity (Supplemental Methods S10). Because many of the MEPs were measured during ongoing motor activity, epochs of EMG data were averaged across trials for a given task, coil position, and orientation, to reduce the effect of ongoing background EMG (i.e., to create an event-related average MEP). No individual trials or MEPs, whether “present” or not, were discarded post hoc without explicitly coding them into the analysis scripts. Four trials were removed in *experiment 4* and 11 trials in *experiment 5*, because of failure of TMS, missed hardware triggers, or experimenter error.

To adjust for between-participant differences in electrode placement, muscle size, or TMS efficacy, before statistical analysis MEPs were rescaled by dividing by the maximum MEP per participant and muscle. To provide an index of, and to display, coil orientation specificity in *experiments 3–5*, rescaled MEP amplitudes were also analyzed in a polar coordinate system. Circular statistics allowed an estimate of the overall orientation preference for each position, resulting in a preferred orientation (i.e., the direction of the resultant vector) and an overall strength of preference (i.e., the length of the resultant vector). For the reported hypothesis tests in *experiment 3*, to adjust for the overall differences in response magnitude across different movement conditions (pegboard, isotonic contraction, rest), these statistics were then recalculated after rescaling the data by the maximum MEP amplitudes per muscle and per condition. This provides a more stringent test of the hypotheses than normalizing across conditions pooled together, since MEP amplitudes, and therefore mean resultant vector lengths, were generally higher during active movement than in other conditions.

Our statistical approach is to make a series of hypothesis-driven comparisons. For MEP data, this involves first comparing each potential MEP amplitude measured after the TMS pulse with a similar epoch before the TMS. Next, these TMS-related EMG differences (MEPs for short) are compared either between experimental conditions (e.g., rest vs. movement) or against zero (e.g., when mapping the presence of MEPs over space). When multiple TMS coil orientations are used (*experiments 3–5*), the data are further processed by computing circular statistics that combine all the tested orientations into a single vector representing the strength and direction of orientation specificity for each TMS location and movement condition. This substantially simplifies and reduces the number of statistical tests required.

EMG and MEPs result in continuous ratio-level data that are appropriately analyzed by standard parametric statistical techniques with samples of size *N* ∼ 12. We have previously verified these assumptions using nonparametric bootstrap resampling methods with samples of *N* ∼ 10 (28). We also ran the same procedure on the smallest dataset included in the present study (*experiment 2b*, *N* = 7) and confirmed that the sampling distributions of the mean were approximately symmetrical and followed an approximate *t*(*n* − 1) or normal distribution (Supplemental Methods S9, Supplemental Fig. S3). We have no reason to assume that the residuals after fitting our simple statistical models will deviate significantly from normally distributed.

In *experiments 3–7*, multiple TMS locations and intensities (5–27) were tested across one to three different conditions. We did not correct for multiple comparisons across locations or intensities in part because mapping data like this will show autocorrelation: the different locations and intensities are not independent from each other. Rather, we relied upon hypothesis-driven tests across multiple independent experiments, focusing on responses with TMS over L-M1-FDI_R_ and L-SMG. Future research should devise appropriate methods for multiple-comparisons corrections that take into account the autocorrelation in spatial or intensity manipulations, for example as used in brain imaging. Further detail is given in Supplemental Methods S8.

In *experiment 7*, MEP latencies were estimated manually by two experimenters (22-yr experience and 1-mo experience, respectively), inspecting the mean MEP traces in custom plots viewed in MATLAB and coming to a subjective, independent judgment about the likely onset. *Rater 1* detected 791 MEPs from 1,344 (58.9%) mean EMG traces; *rater 2* detected 760 (56.6%); both raters detected the same 735 MEPs (54.7%). The estimated latencies for these 735 MEPs had an excellent intraclass correlation coefficient (JASP 0.17) [*r*(733) = 0.958, 95% confidence interval (CI)={0.954,0.962}]. The expert detected a greater number of MEPs and with a lower standard deviation than the novice rater (3.48 ms vs. 3.61 ms), so the expert rater’s latencies were used in the analysis. Using only the second rater’s latencies did not change the conclusions. Following a reviewer’s request, automatic latency estimates were also calculated and are reported in Supplemental Methods S11, Supplemental Fig. S4.

## RESULTS

### *Experiments 1* and *2a*: TMS over L-SMG Impairs Manual Dexterity

**Figure 2.**
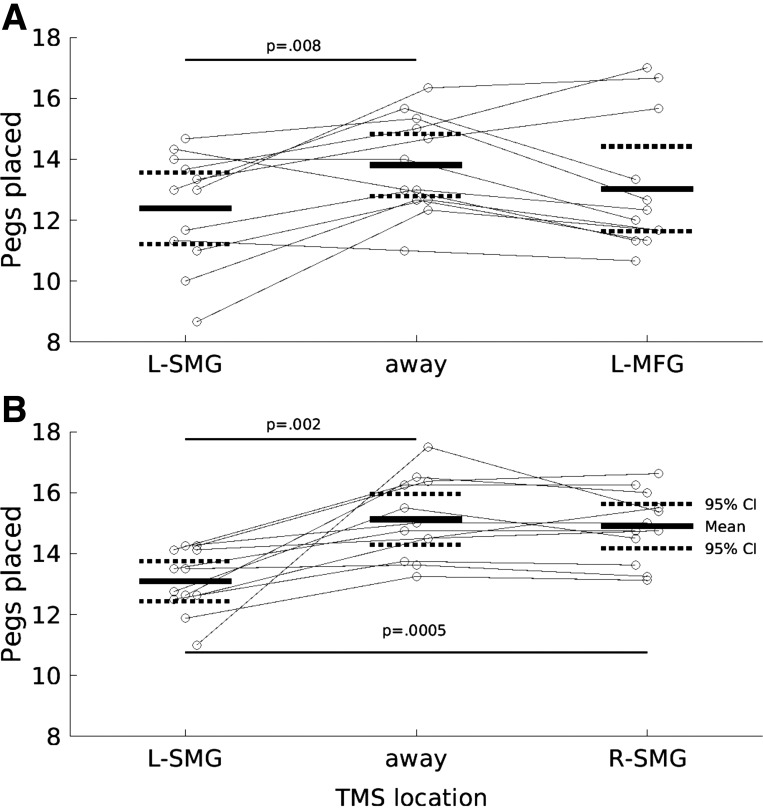
Transcranial magnetic stimulation (TMS, biphasic) over the left supramarginal gyrus (L-SMG) reduces right hand performance on the pegboard task (pegs placed, *y*-axis) compared to TMS away from the head or over a control site selected for its annoyance and discomfort (left middle frontal gyrus, L-MFG). Short horizontal solid and dashed lines show the group means and 95% confidence intervals (CIs). Gray circles and lines show individual participant data. Long horizontal lines and *P* values indicate significant 2-tailed paired *t* tests. *A*: *experiment 1* (*N* = 12): TMS at 1 Hz over L-SMG vs. away from the head and over L-MFG. *B*: *experiment 2a* (*N* = 12), TMS at 2 Hz over L-SMG vs. away from the head and over right (R)-SMG. *P* values relate to paired *t* tests.

One-way ANOVA revealed a significant effect of TMS [*F*(2,22) = 3.97, *P* = 0.034]. During 1-Hz TMS over L-SMG, participants placed a mean ± 95%CI of 12.4 ± 0.54 pegs, which was lower than with TMS away from the head [13.8 ± 0.46, *t*(11) = −3.25, *P* = 0.008] but not significantly different from 1-Hz TMS over the control site, L-MFG [13.0 ± 0.63, *t*(11) = −0.996, *P* = 0.341]. The two control conditions did not differ significantly [*t*(11) = 1.95, *P* = 0.0769] (Fig. 2*A*). During debrief, participants reported that TMS over the control location L-MFG was more painful and annoying than over the experimental location L-SMG, which may explain the lack of a significant difference in number of pegs placed between these two TMS conditions.

*Experiment 2a* aimed to increase statistical power by increasing the effect size: TMS was presented at twice the frequency, task repetitions were increased from three to eight, and the control site (R-SMG) was changed to be closer in subjective annoyance to L-SMG (10). These changes were successful. A significant one-way ANOVA [*F*(2,22) = 16.8, *P* < 0.001] was due to fewer pegs placed with 2-Hz TMS over L-SMG (13.1 ± 0.3) than with both TMS away from the head [15.1 ± 0.38, *t*(11) = −4.03, *P* = 0.002] and TMS over the R-SMG control site [14.9 ± 0.329, *t*(11) = −4.82, *P* = 0.0005]. Pegs placed under the two control TMS conditions did not differ [*t*(11) = 1.04, *P* = 0.321] (Fig. 2*B*).

### *Experiments 2b* and *3*: TMS over L-SMG Evokes MEPs

**Figure 3.**
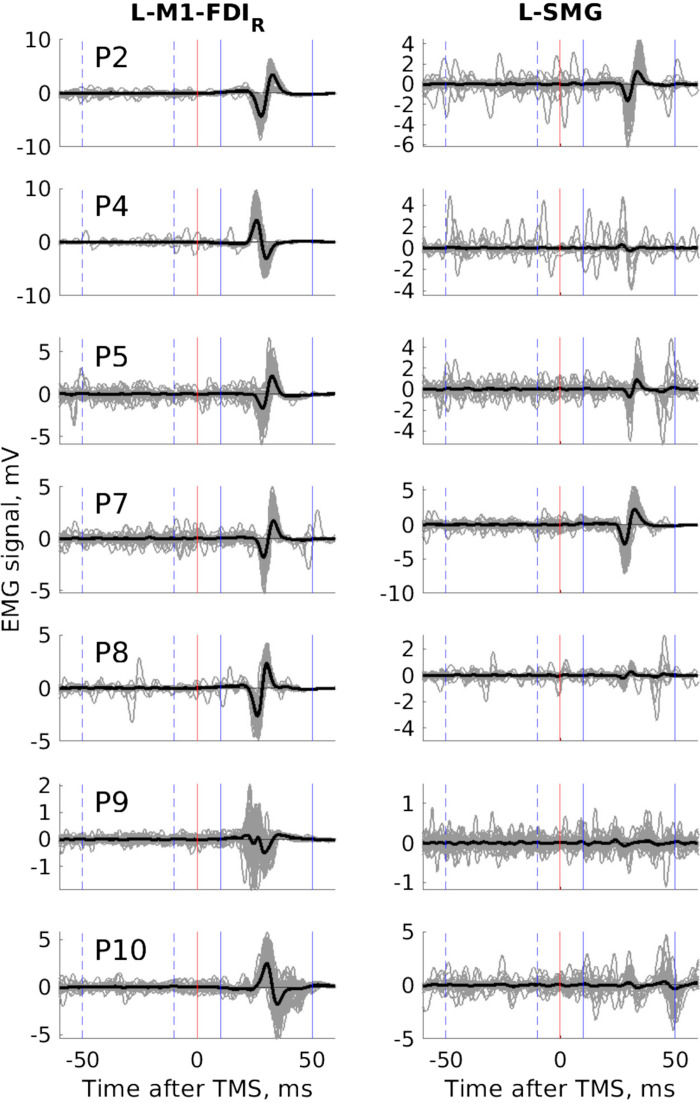
Transcranial magnetic stimulation (TMS, biphasic) over the left supramarginal gyrus (L-SMG) produces motor-evoked potentials (MEPs) in the right first dorsal interosseous (FDI_R_) muscle during the pegboard task (*experiment 2b*, *N* = 7). Each panel shows all 60 raw traces (thin gray lines) and the grand average mean electromyographic (EMG) signal (thick black lines) after TMS over the primary motor cortex (L-M1-FDI_R_, *left*) and L-SMG (*right*) from each participant (from *top* to *bottom*, *P2*, *4*, *5*, *7*, *8*, *9*, *10*). With TMS over SMG, all 7 participants show TMS-related EMG deflections in the 10 ms to 50 ms window following TMS, some participants more than others, all participants more in the post-TMS than the pre-TMS window. The *y*-axis scale varies from a minimum of ±1 mV to ±10 mV.

Anecdotal evidence from debriefing participants in *experiment 2a* suggested that TMS over L-SMG was directly causing, interfering with, or blocking movements of the right hand. To check this, in *experiment 2b* we invited participants to return to complete one 30-s block of the pegboard task during 2-Hz TMS over L-SMG and another with 2-Hz TMS over L-M1-FDI_R_. Seven participants returned. EMG was measured over four hand and arm muscles. Sixty TMS pulses per block were used to create an event-related average MEP. With TMS over L-M1-FDI_R_, all seven participants showed MEPs in all four muscles. More surprising was that all seven participants also showed MEPs in at least three of four muscles with TMS over L-SMG. Because there was no comparison rest condition, we tested this formally by first measuring the 95% confidence interval (CI) of the baseline EMG from −200 to −50 ms, before the TMS pulse. Using a *t* test across participants, we compared the number of time points that were more extreme than this baseline between 10 and 50 ms after TMS (i.e., post-TMS, during the putative MEP) to the same period before TMS (−50 to −10 ms, a pre-TMS control period). This comparison was positive (greater for post-TMS than pre-TMS) in all but one muscle for one participant (i.e., in 27/28 comparisons) and significant across participants for all four muscles [thenar_R_: *t*(6) = 5.49, *P* = 0.002; FDI_R_: *t*(6) = 4.62, *P* = 0.004; flexor digitorum superficialis (FDS_R_): *t*(6) = 3.05, *P* = 0.022; extensor digitorum communis (EDC_R_): *t*(6) = 3.49, *P* = 0.013]. TMS over L-SMG produced low-latency MEPs during the pegboard task (Fig. 3).

**Figure 4.**
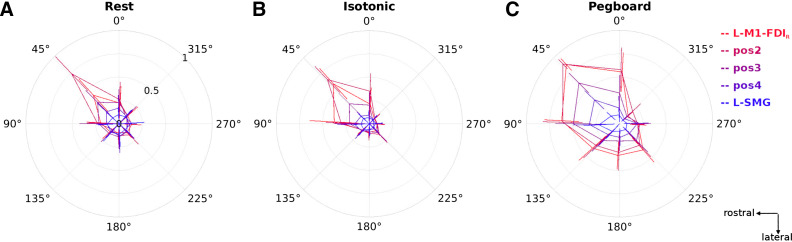
The effect of transcranial magnetic stimulation (TMS, monophasic) coil orientation on motor-evoked potential (MEP) amplitude is strongest during the active movement pegboard task (*experiment 3*). Data show mean and 95% confidence intervals (*N* = 12) of MEP amplitudes (normalized by the maximum MEP amplitude per participant and condition, ranging from 0 to 1, radial axis) for 3 conditions, Rest (*A*), Isotonic contraction (*B*), and Pegboard (*C*); 5 TMS coil positions [red (primary motor cortex, L-M1-FDI_R_) through to blue (left supramarginal gyrus, L-SMG)], and 8 TMS coil orientations (from East at 0°, through North-East at 45°, to South-East at 315°, on polar axes; 90° is toward the nasion). See Supplemental Results S2 and Supplemental Fig. S7 for alternative visualizations of these data along with estimated MEP latency. Data are presented in the same perspective as the participant and brain in Fig. 1.

This post hoc finding was investigated systematically in *experiment 3* by stimulating L-M1-FDI_R_, L-SMG, and three equally spaced intermediate locations while the participant was at rest, during moderate isotonic contraction, and during the pegboard task. MEP amplitude decreased consistently with increasing distance from L-M1-FDI_R_ [*F*(1.81,18.1) = 24.0, *P* < 0.001]. Descriptively, there was no obvious bimodality as a function of distance or an increase in MEPs with TMS over L-SMG; the coil orientation preference of MEPs was strongest with TMS over L-M1-FDI_R_ and decreased with distance from L-M1-FDI_R_ (Fig. 4). The strongest orientation preference was usually the standard North-East direction (45°). Changes in orientation preference with distance appeared similar when measured at rest and during isotonic contraction but different during the pegboard task. Larger MEPs were elicited from a wider range of coil orientations during the pegboard task, and a striking decrease in MEP amplitude was recorded with the coil oriented South (handle anterior) and South-East (handle anterolateral).

**Figure 5.**
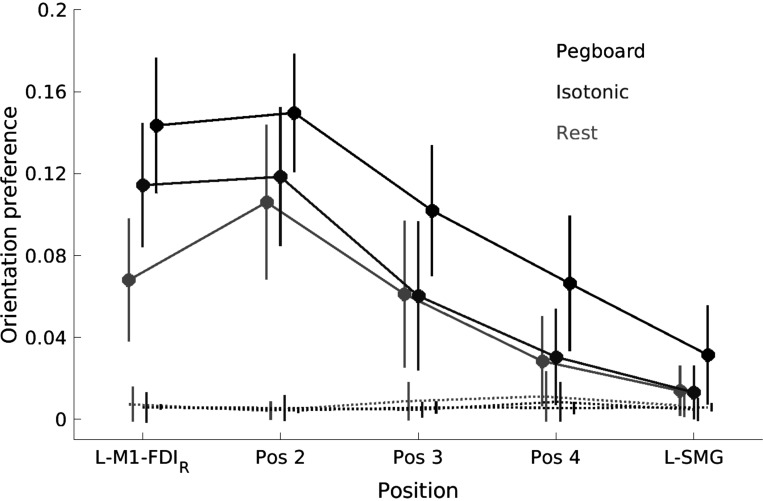
Transcranial magnetic stimulation (TMS, monophasic) coil orientation preference is stronger for the pegboard task compared to both isotonic contraction and rest and remains significantly greater than baseline when the coil is held ∼50 mm away from the primary motor cortex hand area (L-M1-FDI_R_), over the left supramarginal gyrus (L-SMG) (*experiment 3*, *N* = 12). Circles show the mean orientation preference [mean resultant length across 8 orientations of motor-evoked potential (MEP) amplitudes normalized by participant and condition] across 12 participants in 3 conditions (black = Pegboard, mid-gray = Isotonic contraction, light gray = Rest) and for 5 TMS coil positions (between L-M1-FDI_R_ and L-SMG). Error bars show 95% confidence intervals. Solid lines: data measured after the TMS pulse (i.e., from MEPs). Dotted lines: data measured before the TMS pulse (i.e., background electromyographic activity).

To formally test the orientation preferences at different distances from L-M1-FDI_R_ and under different conditions, we calculated the mean resultant vector across all eight coil orientations for each movement condition separately. This analysis asked: How different from circular are the data? The mean resultant vector adds all the individual vectors together and then divides by the number of vectors to give the overall orientation preferences shown in Fig. 4. If MEP amplitudes did not vary with orientation, the mean resultant vector will have length 0. If seven of the amplitudes were zero and one amplitude was positive, the mean resultant vector would have length 1/8. Since there was always some ongoing EMG activity, we subtracted the resultant lengths obtained from EMG activity before the TMS pulse (−50 to −10 ms) from the EMG activity after the TMS (10 to 50 ms; Fig. 5 shows the raw data).

Repeated-measures ANOVA with variables condition (rest, isotonic, pegboard) and position (L-M1-FDI_R_, 2, 3, 4, L-SMG) revealed significant main effects of both position [*F*(2.16,23.8) = 14.4, *P* < 0.001] and condition [*F*(1.35,14.8) = 18.9, *P* < 0.001] but no significant interaction between position and condition [*F*(3.13,34.4) = 1.82, *P* = 0.161]. At all TMS positions, coil orientation preference was stronger in the pegboard condition than during isotonic contraction [significantly stronger with TMS on *position 4*, *t*(11) = 4.41, *P* = 0.001 and over L-SMG, *t*(11) = 2.91, *P* = 0.014] and rest [significantly stronger with TMS over L-M1-FDI_R_ at *position 4*, *t*(11) = 6.68, *P* < 0.001 and over L-SMG, *t*(11) = 2.58, *P* = 0.025]. While orientation preference decreased monotonically with distance from L-M1-FDI_R_ (downward trends in Fig. 5), it was significantly greater than zero at all positions for the pegboard task but only as far as *position 3* for rest and isometric contraction tasks (Fig. 5; significant where the 95% confidence intervals do not include the background mean values, dotted lines). The coil orientation showing the largest MEPs with TMS over L-SMG was North (90°) rather than North-East (45°) (Fig. 4, Pegboard, L-SMG).

Following a reviewer’s request, we tested whether MEP amplitudes were different for the first and second pulses in the pair. There were no significant differences between EMG signals recorded after the first and second pulses, for rest [*t*(11) = −0.247, *P* = 0.810], isotonic contraction [*t*(11) = −0.0563, *P* = 0.956], or the pegboard task [*t*(11) = 0.279, *P* = 0.785] (Supplemental Methods S3, Supplemental Fig. S1).

### *Experiments 4* and *5*: TMS Stimulates Motor Cortex as Far as 50 mm Away

**Figure 6.**
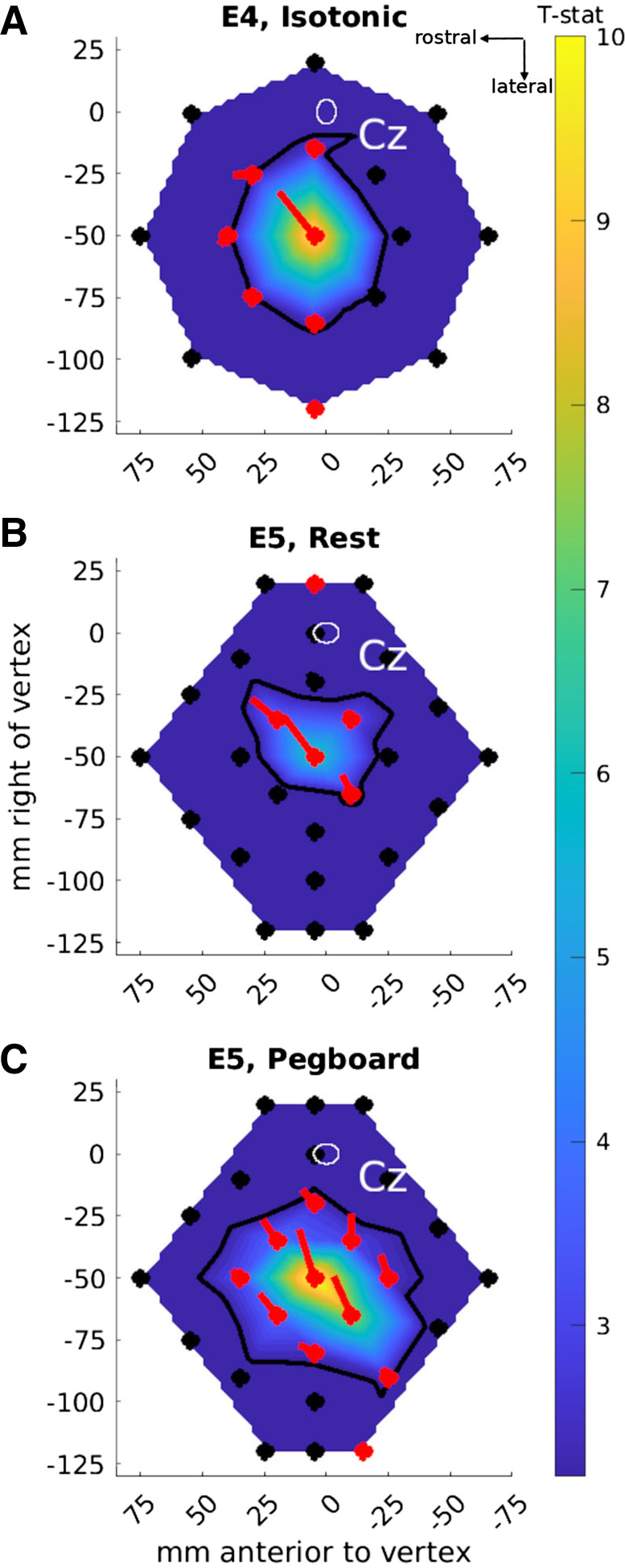
Cartesian maps of motor-evoked potential (MEP) amplitudes (as *t* statistics compared to pre-TMS data, map color) and transcranial magnetic stimulation (TMS, monophasic) coil orientation preferences (oriented lines) in *experiments 4* (*A*; 17 locations, *N* = 12) and *5* (*B* and *C*; 27 locations, *N* = 12). The map background colors show the interpolated *t* statistics comparing the mean MEP amplitudes after vs. before TMS across all stimulated locations [scale bar on *right*; thresholded at *t*(11) > 2.20, *P* < 0.05; nonsignificant *t* statistics are in deep blue]. TMS locations are shown as filled circles. Black symbols: no significant difference between pre- and post-TMS (i.e., no significant MEPs). Red symbols: significant differences [*t*(11) > 2.20, *P* < 0.05] between pre- and post-TMS. The oriented lines show the mean orientation preference vector for each location (red indicates vectors that were significantly greater in amplitude for post- compared to pre-TMS). The thick black contour line shows the interpolated threshold level: TMS presented at locations inside the contour induced significant MEPs. Cz: the white oval shows the mean ± 95% confidence ellipsoid for the origin of the map across participants, at the vertex, Cz[0,0]mm. Data are presented in the same perspective as the participant and brain in Fig. 1.

*Experiment 3* mapped the incidence of MEPs in one dimension: along a line connecting L-M1-FDI_R_ and L-SMG. *Experiments 4–6* mapped MEPs in two dimensions across the scalp. When *experiment 4* was conducted, we did not appreciate the importance of using the pegboard task, so this experiment only involved isotonic contraction, across 17 locations centered on L-M1-FDI_R_ and for four coil orientations (NE, SE, SW, NW). Figure 6*A* shows that significant MEPs (post- vs. pre-TMS) were evoked from L-M1-FDI_R_ and five of the eight “inner” locations 35 mm away but from only one location on the outer circle, 70 mm away. The vectors arising from each location on the map indicate the preferred coil orientation strength and direction: strong and North-East for L-M1-FDI_R_, weak and variable for the surrounding locations. The black contour in Fig. 6*A*, estimating the map area with significant MEPs, covered 3,340 mm^2^.

*Experiment 5* (Fig. 6, *B* and *C*) measured a more homogeneous map and compared rest and pegboard tasks, revealing 4 contiguous locations with significant MEPs during rest (map area 1,890 mm^2^) and 10 contiguous locations with significant MEPs during the pegboard task (map area 4,670 mm^2^). For the pegboard task, 8 of the 10 significant locations had orientation preferences close to North-East. Significant MEPs were evoked from 50 mm away from L-M1-FDI_R_ (*position 12*, 4 cm lateral and 3 cm posterior to L-M1-FDI_R_, approximately over L-SMG (Fig. 6*C*, *bottom right*).

### *Experiment 6*: Remote TMS Effects Are Stronger for a Motor than a Reaction Time Task

**Figure 7.**
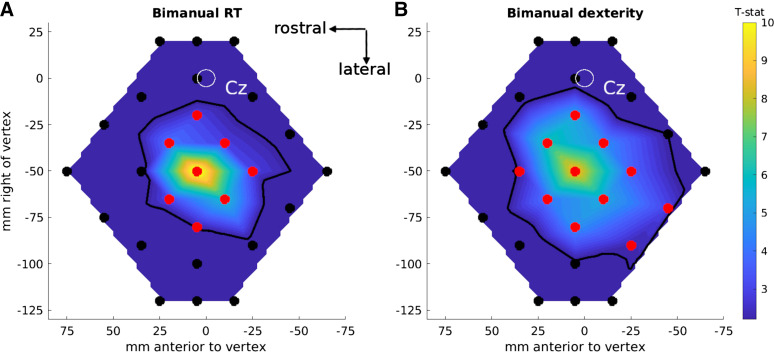
Cartesian maps of motor-evoked potential (MEP) amplitudes as *t* statistics compared to before transcranial magnetic stimulation (TMS, monophasic; data shown as map color) in *experiment 6*: bimanual reaction time (RT) task (*A*) and bimanual dexterity task (*B*) (27 locations each). The map background colors show the *t* statistics comparing the mean MEP amplitudes after vs. before TMS, interpolated across all the locations stimulated [scale bar on *right*; thresholded at *t*(11) > 2.20, *P* < 0.05; nonsignificant *t* statistics are in deep blue]. TMS locations are shown as filled circles. Black symbols: no significant difference between pre- and post-TMS (i.e., no significant MEPs). Red symbols: significant differences (*P* < 0.05) between pre- and post-TMS. The thick black contour line shows the interpolated threshold level: TMS presented at locations inside the contour induced significant MEPs in the hand muscle. Cz: the white oval shows the mean ± 95% confidence ellipsoid for the origin of the map across participants, at the vertex, Cz[0,0]mm. Data are presented in the same perspective as the participant and brain in Fig. 1.

In *experiment 6*, we asked whether MEPs could be evoked from L-SMG during a bimanual RT task or only during a bimanual dexterity task. During the RT task (Fig. 7*A*) significant MEPs were evoked from eight locations (map area 3,990 mm^2^), whereas during the bimanual dexterity task (Fig. 7*B*) MEPs were evoked from 11 locations, including 2 (*positions 11* and *12*) over the inferior parietal lobule (map area 6,450 mm^2^). MEP amplitude was larger at these IPL positions during the bimanual dexterity task than during the bimanual RT task [significant for *position 11*, *t*(11) = 2.41, *P* = 0.035, but not *position 12*, *t*(11) = 1.71, *P* = 0.11].

### *Experiment 7*: MEP Amplitude and Latency Do Not Support Separate Sources

**Figure 8.**
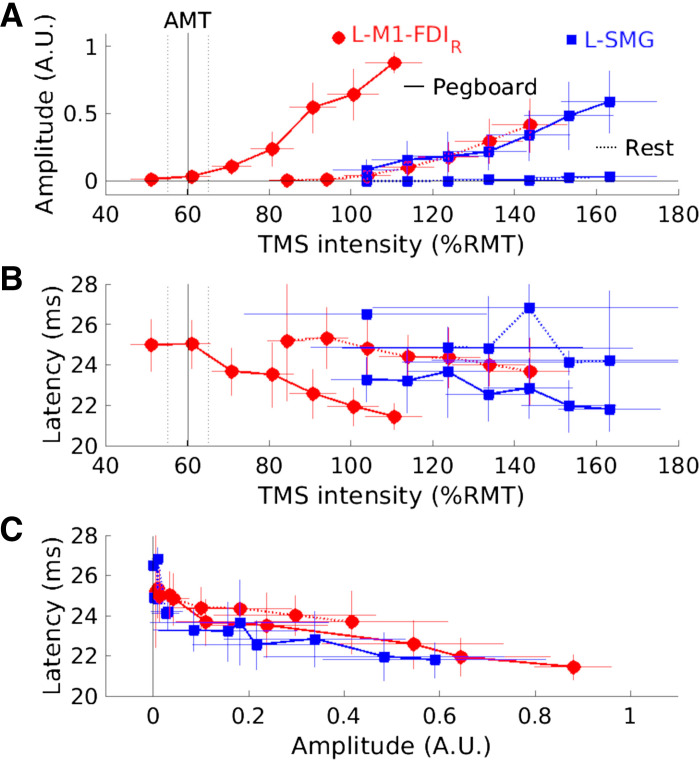
Motor-evoked potential (MEP) amplitude and latency across 7 transcranial magnetic stimulation (TMS, monophasic) intensities provide no evidence for separate sources of MEPs evoked from TMS over left motor cortex hand area (L-M1-FDI_R_) or left supramarginal gyrus (L-SMG; *experiment 7*, *N* = 11). TMS was delivered over L-M1-FDI_R_ (red circles) and L-SMG (blue squares) during rest (dotted lines) and the pegboard task (solid lines). Thin red and blue horizontal and vertical lines show 95% confidence intervals for the means, based on different numbers of participants for each data point. Vertical black lines in *A* and *B* indicate the mean (solid) and 95% confidence intervals (dotted) active motor threshold (AMT) during the pegboard task (AMT_pegboard_). *A*: normalized MEP amplitude [*y*-axis, arbitrary units (A.U.)] vs. TMS intensity [*x*-axis, % of resting motor threshold (%RMT_Rossini94_)]. *B*: MEP latency (*y*-axis, ms) vs. TMS intensity (*x*-axis, %RMT_Rossini94_). *C*: MEP latency (*y*-axis, ms) vs. normalized MEP amplitude (*x*-axis, A.U.). The generally overlapping curves in *C* are consistent with a single neural source of the MEPs, evoked at lower TMS intensities from directly over L-M1-FDI_R_ and at higher intensities over L-SMG, 55 mm away on the scalp.

We collected recruitment curves for 11 participants under four conditions: during the pegboard task and at rest with the TMS coil centered over L-M1-FDI_R_ or over a location 55 mm away from L-M1-FDI_R_ (∼39 mm lateral, ∼39 mm posterior), likely over the L-SMG. Figure 8 shows the mean MEP amplitudes, manually estimated mean MEP latencies, and the relationships between amplitude and latency for seven TMS intensities per condition. During the pegboard task, TMS over L-M1-FDI_R_ evoked MEPs significantly above zero at a mean (SD) of 61.0 (7.2)%RMT_Rossini94_, and MEP amplitude increased without reaching a plateau up to the maximum intensity of 111 (10.4)%RMT_Rossini94_. At rest, TMS over L-M1-FDI_R_ evoked significant MEPs at and above 94.2 (7.4)%RMT_Rossini94_. With TMS over L-SMG, significant MEPs were evoked during the pegboard task from 104 (12.0)%RMT_Rossini94_ but not at any intensity while at rest [up to 163 (17.5)%RMT_Rossini94_; Fig. 8*A*].

MEPs latencies decreased with increasing TMS intensity, from a mean (SD, *N*) of 25.2 (4.2, *N* = 5) ms to 23.7 (2.3, *N* = 11) ms for TMS over L-M1-FDI_R_ at rest and from 25.1 (1.9, *N* = 5) ms to 21.5 (1.0, *N* = 11) ms during the pegboard task. Latencies for TMS over L-SMG were similar, from 26.5 (not applicable, *N* = 1) ms to 24.2 (1.4, *N* = 6) ms at rest and from 23.3 (1.6, *N* = 7) ms to 21.8 (1.3, *N* = 10) ms during the pegboard task (Fig. 8*B*).

With different numbers of MEPs detectable for different participants and conditions, a full statistical analysis was not possible. However, the four strongest TMS intensities evoked MEPs in at least 10 participants during the pegboard task for both TMS sites, so two pairs of TMS intensities that produced the most similar MEP amplitudes were selected for analysis. TMS *intensity 6* over L-M1-FDI_R_ evoked mean (SD) MEP amplitudes of 0.610 (0.276) arbitrary units (A.U.), which were not significantly different from TMS *intensity 7* over L-SMG [0.648 (0.306) A.U., *t*(9) = −0.272, *P* = 0.792]. The latencies for these MEPs did not differ significantly [L-M1-FDI_R_ = 21.8 (1.4) ms, L-SMG = 21.8 (1.3) ms, *t*(9) = −0.07, *P* = 0.943]. Similarly, TMS *intensity 4* evoked MEP amplitudes that did not differ significantly between the sites [L-M1-FDI_R_ = 0.247 (0.201) A.U., L-SMG = 0.237 (0.195) A.U., *t*(9) = 0.09, *P* = 0.929]. The MEP latencies did not differ significantly [M1 = 23.1 (2.1) ms, L-SMG = 22.6 (1.8) ms, *t*(9) = 0.696, *P* = 0.504]. Overall, the latency-amplitude curves and their 95% confidence intervals overlapped considerably, providing no evidence for a separate anatomical source for MEPs evoked by TMS over the two sites (Fig. 8*C*).

### Across-Experiment Analysis of Experiments *2b*, *3*, *5*, *6*, and *7*

During natural movement, TMS over M1 and nearby locations resulted in large MEPs. For example, with TMS over SMG (∼50 mm away from M1), the mean raw MEP amplitudes ranged from 0.47 mV (*experiment 6*) to 1.64 mV (*experiment 2b*), with an across-experiment mean of 0.78 mV. MEP amplitudes, effect sizes, and a power analysis for selected TMS locations and conditions are presented in Supplemental Results S3, Supplemental Table S2. Effect sizes for detecting MEPs in *experiments 2b*, *3*, *5*, *6*, and *7* (i.e., for TMS over SMG during natural movement) were all large. A meta-analysis across these five selected effects yielded a combined effect (Cohen’s *d*) of 0.76. This is a large effect size. Using this effect size and repeating only the very simplest aspect of our methods, 10 trials with TMS over SMG, future studies would need to recruit 14 participants per study to replicate this effect with an 80% probability of achieving *P* < 0.05.

Across these five experiments, there were no significant correlations between the participant’s RMT and the critical effect size comparing the amplitude of EMG signals after versus before TMS, when TMS was over L-SMG during natural movement [*experiment 2b*: *r*(5) = 0.480, *P* = 0.276; *experiment 3*; *r*(10) = 0.035, *P* = 0.915; *experiment 5*: *r*(10) = 0.0119, *P* = 0.971; *experiment 6*: *r*(10) = −0.0369, *P* = 0.909; *experiment 7*: *r*(9) = 0.113, *P* = 0.727]. Combining all the data revealed no overall correlation between RMT and effect [*r*(52) = 0.077, *P* = 0.582] (Supplemental Results S4, Supplemental Table S3, and Supplemental Fig. S8).

### *Experiment 8*: TMS over Parietal and Premotor Cortices Evokes MEPs during Movement

**Figure 9.**
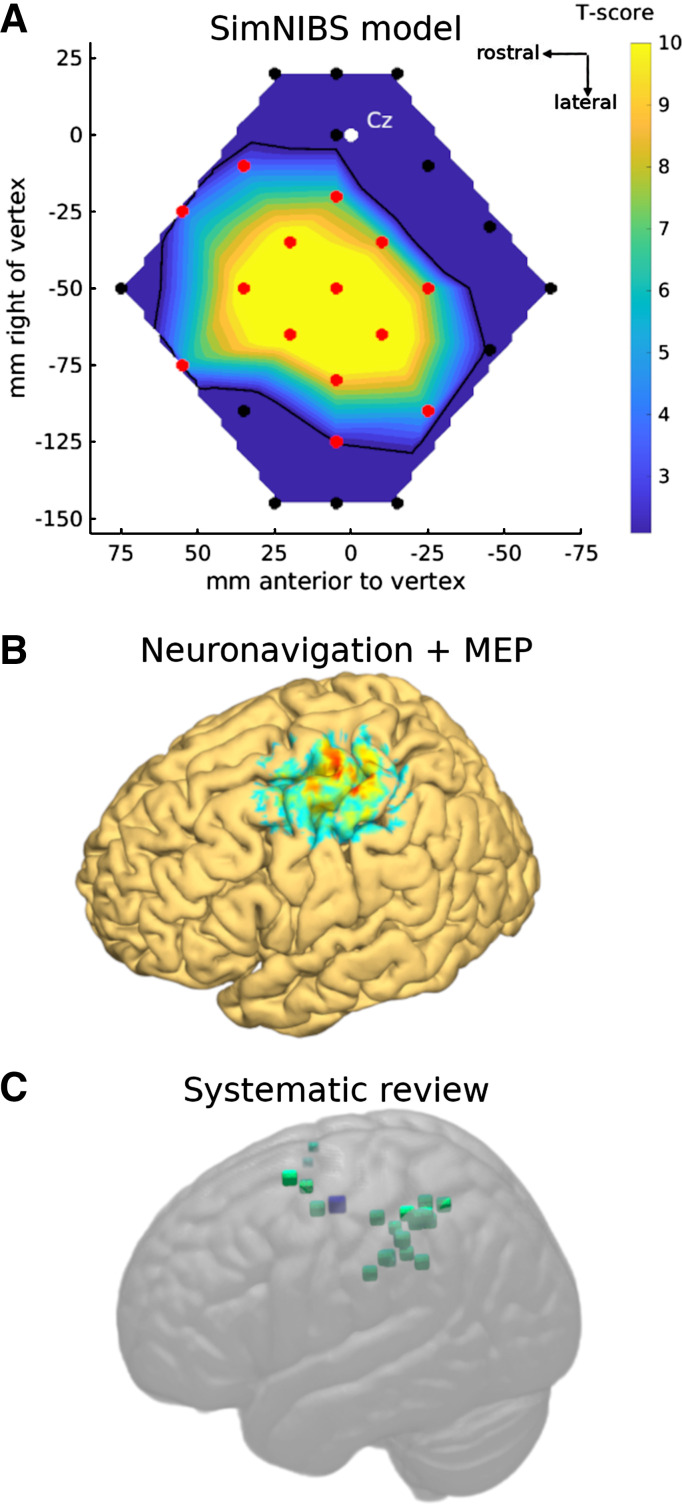
Modeling and localizing the scalp and brain areas over which transcranial magnetic stimulation (TMS) can generate motor-evoked potentials (MEPs) in hand muscles during hand movement. *A*: SimNIBS modeling of the electric field generated by a TMS pulse at 110% resting motor threshold in the North-East orientation. The data show the expected activation of the motor cortex hand area from each location, thresholded at the estimated cutoff where MEPs were detected (Supplemental Fig. S3) and expressed as a *t* test against the threshold value. The SimNIBS modeling data reflect the empirical findings (Figs. 6 and 7). *B*: 3-dimensional (3-D) rendering of MEP amplitudes obtained from 7 participants with neuronavigation data. The image shows an approximate, conservative representation of which brain areas, when targeted with TMS, are likely close enough to the hand area of the left motor cortex to induce MEPs during right hand and arm movement. Voxel intensity is a weighted sum proportional to the inverse of distance between each voxel and all 27 TMS sites, thresholded, rescaled, transformed to MNI space, smoothed and averaged across participants, and rendered with FieldTrip and FSL tools. *C*: systematic review revealed 19 studies that targeted 41 locations (small green squares) away from, but within 42 mm of, the left primary motor cortex hand area (MNI[−38,−15,58]mm, large blue square). Fewer than 41 distinct locations are visible since some locations were stimulated multiple times across studies. Data are presented in the same perspective as the participant and brain in Fig. 1.

We used Brainsight neuronavigation, FSL, and SimNIBS electrical field modeling (24) in 22 participants to estimate where on the scalp, and over which brain areas, TMS evokes MEPs in FDI_R._ Across locations at the group level, the simulated electric current strength correlated reasonably well with mean FDI_R_ MEP amplitudes from *experiment 5* (Supplemental Fig. S3). Systematically varying the threshold for MEPs (from 0 to 79 V/m, i.e., excluding progressively more data points with low modeled current) revealed optimal MEP-current correlations at a mean threshold of 31.1 V/m across four coil orientations and two conditions. This corresponded to 32.6% of the maximum value of volts per meter obtained across all participants and positions. This threshold was used to create, in Fig. 9*A*, a map of the mean modeled electrical currents within a 11 × 11 × 11-mm voxel centered on L-M1-FDI_R_. This simulated map matches quite well the empirical results from *experiments 5* and *6*, except that the simulated map is larger (9,370 mm^2^), resulting in 14 stimulated locations having a modeled current significantly above the estimated MEP threshold. This is likely because the SimNIBS modeling does not take into account the orientation sensitivity of the motor cortex (Fig. 4). MNI coordinates for all 27 locations stimulated are provided in Supplemental Tables S4, S5, and S6. The correlations between MEP amplitude and estimated distance to L-M1-FDI_R_ were *r*(25) = −0.85 and −0.84 (both *P* < 0.001) in *experiments 5* and *6*, respectively.

For seven participants with both MEP and neuronavigation data available, a 3-D rendering of the brain locations over which TMS reliably evoked MEPs from L-M1-FDI_R_ is given in Fig. 9*B*. This provides an approximate and conservative estimate of the cortical locations that, when targeted with TMS, are close enough to L-M1-FDI_R_ to result in MEPs being evoked during hand movement.

### Systematic Review: Studies with TMS near Motor Cortex during Hand Movement

**Figure 10.**
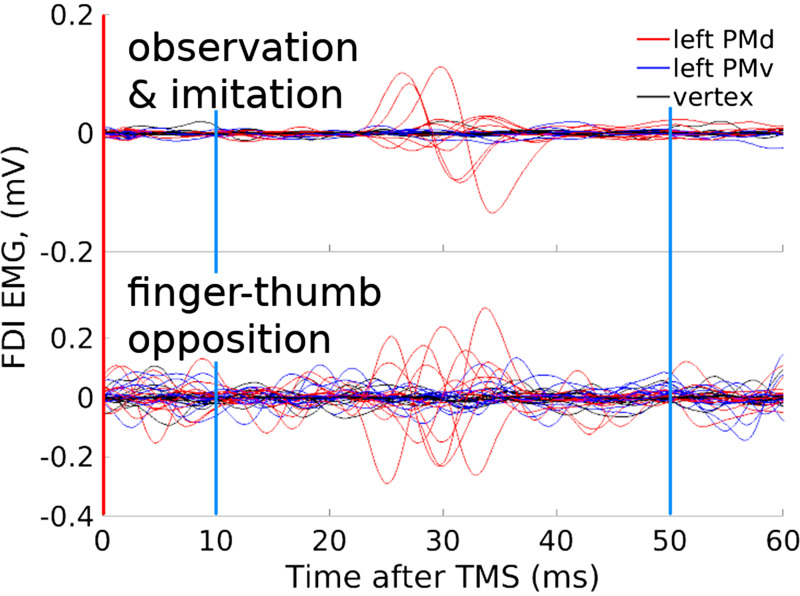
Motor-evoked potentials (MEPs) were elicited with transcranial magnetic stimulation (TMS) over premotor cortex during movement in a previously unanalyzed dataset. Red vertical lines show the onset of TMS; blue vertical lines show the window from 10 ms to 50 ms after TMS in which MEPs in the hand and arm will occur. The data are from first dorsal interosseus (FDI) muscle activity during action observation, imitation, and finger-thumb opposition and after TMS at 110% distance-adjusted resting motor threshold (RMT) [Stokes et al. (32)] over dorsal (PMd, red) and ventral (PMv, blue) premotor cortex and vertex (black). Grand average EMG traces from 1,176 TMS pulses during observation and imitation (*top*) or 15 pulses during finger-thumb opposition movements (*bottom*) for each of 12 participants; previously unpublished data from the study by Reader and Holmes (31). Further examples are provided in Supplemental Results S11.

In 30 of 50 reviewed experiments EMG data were used, for example, to define RMT. In many of these experiments it was reported that MEPs were not observed during the task or in pre- or postexperiment checks or controls. However, only 2 of the 30 experiments with EMG data included any analyses examining MEPs or other EMG variables such as silent periods. Both of these experiments clearly reveal significant corticospinal activation with TMS over the premotor cortex during handwriting (Fig. 4 in Ref. 17) or arm reaching (Fig. 11 in Ref. 29). In one additional paper studying sequential finger tapping movements, MEPs were not analyzed but seem to be present in the single-participant example EMG data that were shown (Fig. 2 in Ref. 30). Analysis of the previously unanalyzed EMG data from our own work (31) confirmed the presence of MEPs in at least six participants’ FDI and brachioradialis muscles when TMS was presented over the dorsal premotor cortex during action observation, imitation, and finger-thumb opposition (Fig. 10).

To estimate which published experiments might contain muscular artifacts from direct M1 stimulation, we first estimated the distance between the reported TMS locations and the hand area of the motor cortex. Our neuronavigation data showed that SMG, over which TMS consistently evoked MEPs, is a mean (SD) of 33 (5.7) mm lateral and 35 (7.8) mm posterior to L-M1-FDI_R_ hand, or ∼49 (6.7) mm in a direct line along the scalp. Our targeted MNI coordinates for L-M1-FDI_R_ and L-SMG within the brain were 37 mm apart (M1 = MNI[−38,−15,58]; SMG = MNI[−57,−44,44]). Our mapping experiments found that significant MEPs were evoked with the coil positioned up to 55 mm away on the scalp, so this cutoff was used, corresponding to 42 mm within the brain (Fig. 9*C*).

Across the 50 reviewed experiments, 96 target locations were provided in 3-D MRI coordinates and 21 in 2-D scalp-based coordinates. Of the 117 target locations, 54 were close to the hand area of the primary motor cortex during active hand movement (derived from 22 articles reporting 26 experiments; Supplemental Table S3, Supplemental Fig. S4), 41 were within 42 mm in the brain [mean (SD) = 30.2 (6.6) mm], and 13 were within 55 mm on the scalp [mean (SD) = 31.4 (11.6) mm] from the M1-hand area. The targeted brain areas included supramarginal and angular gyrus, superior parietal lobule, anterior and middle intraparietal cortex, dorsal and ventral premotor cortex, supplementary motor area, and superior frontal sulcus. A brief attempt to meta-analyze the effect sizes across studies was abandoned because of the considerable heterogeneity of purpose, design, and dependent variables across these studies (see Supplemental Discussion S2 for further details).

## DISCUSSION

### Summary

Across seven experiments, transcranial magnetic stimulation (TMS) over the left supramarginal gyrus (SMG) interfered with right hand dexterity and evoked motor-evoked potentials (MEPs). When the TMS coil was over left SMG, coil orientation significantly influenced MEP amplitude only during the natural goal-directed movement task of pegboard manipulation and not during rest or simple isotonic contraction. The latency and amplitude of MEPs following TMS over SMG were consistent with those following TMS over M1. We conclude that TMS over SMG and M1 stimulated the same cortical source. Electric field modeling supports our finding that MEPs can be generated from as far as 50–55 mm away on the scalp from M1, based on a simulated electrical current threshold that seems to be ∼31 V/m for generating MEPs from M1 during natural movement. Systematic review identified a large number of previous experiments using online TMS sufficiently close to M1 to generate MEPs during natural movement. Below we discuss the effective spatial resolution of TMS, how natural movement engages motor cortex much more than isotonic contraction, how response magnitude, response latency, and coil orientation preference can be used to distinguish cortical sources, and which other TMS studies might be affected by muscular artifacts.

### TMS Is Not as Focal as Many Researchers Seem to Have Assumed

TMS is often described as a “focal” technique, with an effective stimulation radius of perhaps ∼5–20 mm and a depth of ∼20 mm (e.g., Ref. 33). We agree that this applies when participants are at rest and MEPs are recorded after stimulation of the motor cortex. Our work shows that this does not apply during natural movement. When a participant is at rest, with their muscles relaxed, it can be difficult to find the optimal scalp location to generate MEPs in their hand and arm. The coil needs to be moved around, and the intensity of the TMS increased, until MEPs are found. In most participants, changing these two parameters is sufficient. But some participants have resting thresholds as high as 70%MSO or more. We often asked these participants to make a slight isotonic contraction in their hand to facilitate finding the optimal location and intensity. In the present work and in some unpublished experiments, we have noticed that isotonic contraction only relatively weakly modulates the excitability of M1 compared, for example, to active, dynamic reaching, grasping, and manual dexterity tasks.

Under these conditions of natural, goal-directed movement, we found that motor thresholds were much, much lower than resting thresholds: 61% of RMT_Rossini94_ on average and as low as 21%MSO in one participant. This is much lower than we had expected. The corollary of much lower thresholds is that lower TMS current is required to generate MEPs. Given that the magnetic field strength decreases with distance, and given a constant TMS intensity, TMS near M1 will therefore be effective at larger distances during natural movement. We found that at 110% of resting motor threshold, the most commonly used TMS intensity in the reviewed movement studies, we could evoke MEPs from 50–55 mm away on the scalp during natural movement. In their extensive review of TMS methodology and experimental logic, Bergmann and Hartwigsen (34) described the amplitude of MEPs evoked during a slight precontraction of a muscle compared to when at rest as “an impressive example for state dependency” (p. 211). State dependency refers to how the effects of TMS depend on what the participant is doing and their ongoing brain state, both in target and nearby nontarget brain areas. Our data suggest that isotonic contraction only weakly modifies the state of the primary motor cortex relative to rest, whereas performance of a meaningful, natural manual dexterity task is a yet more impressive example of state dependency in TMS (Figs. 4 and 5; Supplemental Fig. S7b).

The focality of TMS is not fixed but varies according to coil position and orientation, the shape of the participant’s brain, and the context in which the brain is stimulated (18, 23).

### Corticospinal Excitability Is Much Higher during Natural Movement

The effect of natural movement on corticospinal excitability has surprised us. Based on our measures of corticospinal excitability, the motor cortex seems much more highly engaged, and its orientation sensitivity very different, during natural movement. The polar plots of the coil orientation sensitivity (Fig. 4) showed that during rest and isotonic contraction motor cortex was sensitive mostly to TMS between 0° and 90° (East to North), with the coil handle pointing laterally to posteriorly, as is well known (35). By contrast, during the pegboard task there was a striking change in the overall shape and degree of orientation sensitivity, including both an overall increase in orientation preference as well as a profound decrease of MEP amplitudes with coil orientations of 270–315° (South and South-East), with the handle pointing anteriorly and anterolaterally, respectively (Figs. 4 and 5; Supplemental Fig. S7). Coil orientation interacts with TMS intensity in its effects on corticospinal excitability in resting muscles (36). Likely because of nonlinearities in the input-output response properties of motor cortex, at low TMS intensities the canonical North-East orientation was optimal for inducing MEPs. As TMS intensity increased from 110% to 140%RMT, MEPs were elicited at a much wider range of orientations (36). How orientation sensitivity and TMS intensity interact during isometric contraction and natural movement may be worth further investigation.

Profound changes in corticospinal excitability during natural movement are not new [Lemon et al. (23)]. In their study, TMS during reach, grasp, and lift movements generated MEPs that were greatly, and nonlinearly, modulated by movement phase, being maximal when the participant’s hand made contact with the target object. These authors found that MEP modulation often did not correlate with ongoing background EMG activity, and the strongest modulations occurred with TMS intensities below each participant’s resting motor threshold. During natural movement, the motor cortex is commanding the muscles and driving corticospinal excitability much more than during rest, and in ways and to extents that may not be predicted by resting motor thresholds alone. The organization of the motor cortex has been rethought, not as a map of individual muscles but as a command-and-control center for natural, ethological movement (37, 38). Beyond the motor cortex, the same conclusions must apply: TMS presented away from the motor cortex can still have profound effects on the corticospinal system, even at low stimulation intensities, if natural movements are ongoing. If there are similar changes in excitability thresholds for brain regions outside of the primary motor cortex, the implications for TMS studies could be wide-ranging.

### Response Magnitude, Latency, and Coil Orientation Indicate Specific Corticospinal Pathways

Across our experiments, we were unable to rule out the possibility that the short-latency MEPs generated from TMS over SMG were generated in M1, for four reasons.

First, MEP amplitudes decreased consistently with distance from M1. They remained significantly larger than the pre-TMS baseline with TMS over SMG but no further than when the coil was 55 mm along the scalp from M1. Our 1-D (*experiment 3*) and 2-D (*experiments 4*, *5*, and *6*) maps and our 3-D modeling of the expected induced current were all consistent with a single source of MEPs in M1, regardless of where the TMS coil was placed. There was no apparent bimodality in MEP amplitude as a function of coil location between M1 and SMG or in the 2-D maps.

Second, when the amplitude of MEPs generated by TMS over M1 and SMG was matched, their latencies did not differ significantly (Fig. 8*C*). If MEPs were generated from different cortical sources, one way to distinguish them is by comparing their latencies (39, 40). It is of course possible that two cortical sources could produce very similar amplitude-latency curves, but parsimony requires that we accept the simplest hypothesis that is compatible with our evidence.

Third, the coil orientation specificity decreased consistently with distance from M1. There was no apparent bimodality in orientation preference as a function of coil location between M1 and SMG (Fig. 5).

Fourth, the preferred coil orientation was always between North and East, usually, and on average, at the canonical North-East orientation, with the coil handle pointing posterolaterally. This remained true when TMS was presented over SMG (Figs. 4 and 6).

One puzzle remains: While we focused on SMG in *experiments 1*, *2*, *3*, and *7*, we also stimulated scalp locations equally distributed around M1 in *experiments 4*, *5*, and *6*. So why was it that the most distant effects on M1 were obtained with TMS over SMG but not, for example, from locations equally distant from M1 but in other directions? We suspect that this is due primarily to the orientation sensitivity of the hand area of M1. The optimal coil orientation for the hand area is North-East, or ∼45°, with the handle pointing posterolaterally, inducing a posterior-to-anterior current in the motor cortex (41). When the coil was positioned over SMG, the North-East orientation aligned with M1, so the induced current direction (posterior to anterior, starting posterior to M1) was likely still sufficient for stimulating M1. By contrast, with the coil positioned at a location North-East from M1 (*position 3* in Fig. 1*H*, over the superior frontal gyrus and/or dorsal premotor cortex; Supplemental Table S2), the induced current could flow either posterior to anterior or anterior to posterior depending on coil orientation, but we assume that it always starts anterior to M1. This position seems less able to excite corticospinal neurons in the motor cortex.

### Parietal and Premotor TMS Studies of Movement Are Likely Affected by Muscular Artifacts

In at least 54 published experimental manipulations where TMS was targeted outside of primary motor cortex during an active hand and/or arm movement task, the TMS coil was likely close enough to the hand and arm area of M1 to evoke MEPs in at least some muscles, in at least some participants. The average TMS coil size (∼70 mm), shape (figure of 8), and intensity (∼110%RMT) in the previous experiments were very similar to those used in our work. It is very likely that at least some of these previous experiments were affected by (trivial) activations of M1, resulting in MEPs, muscle twitches, or the “blocking” of movements, as reported by our own participants and others (17). Of 30 reviewed studies that recorded EMG data, only 2 reported any analysis of MEPs, and both found significant evidence for them (17, 29). It is an open question whether, or how, the dependent variables of most interest in these movement studies were affected by MEPs evoked in participants’ muscles, following stimulation at these 54 locations. Although those studies using EMG typically recorded only a single muscle, and although the present study focused on the hand and on hand movements, TMS within ∼55 mm of any part of the motor cortex has the potential to interfere with movement of any part of the body. Researchers need to rule out the potential effects of unintended motor stimulation on their primary dependent variables.

The magnetic field and/or electric currents generated under typical conditions are sufficient to evoke MEPs from as far as 55 mm away from M1. This has implications not only for online TMS studies of active movement (where MEP artifacts can easily be measured) but also for all TMS studies. Although researchers may target TMS to particular brain regions, direct effects on brain areas up to 55 mm away (as measured on the scalp) or 42 mm away in the brain cannot be discounted, at typical stimulation levels of ∼105%RMT or higher.

Our work also has implications for TMS studies using two coils to examine one brain area’s connectivity with M1 (e.g., Ref. 42). The first TMS pulse presented away from M1 may be sufficiently strong to stimulate M1 directly, such that if followed by a second TMS pulse over M1 between 1 and 7 ms later, it might induce the well-studied short-latency intracortical inhibition (SICI; Ref. 43), decreasing the amplitude of MEPs evoked by the subsequent TMS pulse applied directly over M1. Similarly, for TMS pulses presented over the motor cortex on the opposite side (e.g., right M1) of the head, if the intensity is strong enough it may stimulate contralateral motor cortex directly (i.e., left M1), evoking so-called ipsilateral MEPs or ipsilateral silent periods in the right hand. Ipsilateral MEPs are known to require high TMS intensities and contraction of the ipsilateral muscles (44). Data relating to these questions will be reported elsewhere.

### Limitations

Our central finding is that TMS as far as 55 mm away on the scalp, targeting brain areas as far as 42 mm away from M1, reliably generates large MEPs at typical levels of stimulation during natural movement. What we cannot claim is whether these levels of motor activation are necessary and/or sufficient to interfere with ongoing movement. Although we measured TMS-related impairments in manual dexterity in *experiments 1* and *2a*, all subsequent experiments focused on measuring MEPs rather than movement; the experiments were not designed or executed with movement performance in mind. When recordings of movement performance were taken, they were occasionally incomplete and quite variable and did not produce as clear, or as statistically significant, effects as our primary outcome variable. We suspect that the presence of MEPs in the muscles involved in hand movement does indeed interfere directly with performance, but our experiments and data do not allow this direct conclusion.

Our studies focused on manual dexterity tasks, the hand area of primary motor cortex, and EMG activity recorded from hand and arm muscles. We suspect that the implications of our work will extend most directly to TMS studies of M1 (e.g., to face and leg areas of M1) and to TMS studies of natural movement (e.g., targeting premotor and parietal cortex). We can only speculate whether or how other cortical areas or outputs might be affected by the experimental manipulations used here.

### Recommendations

One part of the solution to the problem of unintended motor activation, at least in studies of movement, is to record EMG from muscles that are relevant to the experimental task, to check for MEPs in every participant, and check whether MEPs correlate with the study’s primary outcome variables (e.g., Ref. 45). This must be done during the full movement or the task itself, rather than before or after the experiment, or when the participant is at rest or maintaining an isotonic contraction. Movement context has massive effects on corticospinal excitability (23) and therefore the likelihood that TMS will interfere with ongoing movement. It is possible, and likely, that using a smaller coil will reduce the potential for stimulation of M1 from remote areas of the scalp. We did not have access to a smaller coil so could not assess this empirically. We recommend that others do so. In nonmovement studies, or those stimulating locations further from M1, alternative methods are required to ensure that TMS targets only the intended brain area (18, 34).

### Conclusions

During natural movement, the corticospinal motor system is much more excitable than during isotonic contraction or rest. This increased corticospinal excitability results in lower thresholds for MEPs. Lower MEP thresholds mean that a TMS pulse presented from further away on the scalp can have the same effect as a closer TMS pulse at rest or during isotonic contraction. Under typical TMS conditions of monophasic stimulation at 110% of the resting motor threshold with a 70-mm figure-of-eight coil, we evoked significant MEPs with TMS positioned over the supramarginal gyrus. This location was 49–55 mm away on the scalp from the optimal location for M1 stimulation. This finding was replicated in five independent experiments. We conclude that movement context dramatically changes the effective focality of TMS for evoking MEPs. The implications for TMS studies more generally need to be evaluated.

## DATA AVAILABILITY

Source data for this study are openly available at https://osf.io/2xytm.

## SUPPLEMENTAL MATERIALS

Supplemental Methods S1–S11, Supplemental Results S1–S11, Supplemental Discussions S1 and S2, Supplemental Figures S1–S11, and Supplemental Tables S1–S7: https://osf.io/6zmy3.

Systematic Review: PubMed search results: https://osf.io/wpy3t.

Systematic Review: PubMed papers details: https://osf.io/9sa3y.

Systematic Review: reference papers details: https://osf.io/meqfp.

Systematic Review: included articles: https://osf.io/ce9kv.

## DISCLOSURES

No conflicts of interest, financial or otherwise, are declared by the authors.

## AUTHOR CONTRIBUTIONS

N.P.H., E.M.C., B.M., K.G., D.G., T.J., A.V.L., and E.S.P. conceived and designed research; N.P.H., N.V.D., E.M.C., B.M., K.G., D.G., T.J., A.V.L., E.S.P., and S.Z. performed experiments; N.P.H., E.M.C., B.M., K.G., D.G., T.J., A.V.L., and E.S.P. analyzed data; N.P.H., E.M.C., B.M., K.G., D.G., T.J., A.V.L., E.S.P., and A.T.R., interpreted results of experiments; N.P.H. and E.M.C. prepared figures; N.P.H. drafted manuscript; N.P.H., N.V.D., E.M.C., B.M., K.G., D.G., T.J., A.V.L., E.S.P., S.Z., and A.T.R. edited and revised manuscript; N.P.H., N.V.D., E.M.C., B.M., K.G., D.G., T.J., A.V.L., E.S.P., S.Z., and A.T.R. approved final version of manuscript.
